# Development of an integrated and inferenceable RDF database of glycan, pathogen and disease resources

**DOI:** 10.1038/s41597-023-02442-2

**Published:** 2023-09-06

**Authors:** Koichi Arakawa, Tamiko Ono, Kiyoko F. Aoki-Kinoshita, Yasunori Yamamoto

**Affiliations:** 1 Faculty of Science and Engineering, Soka University, 1-236 Tangi-machi, Hachioji City, Tokyo, 192-8577 Japan; 2 Glycan and Life Systems Integration Center (GaLSIC), Soka University, 1-236 Tangi-machi, Hachioji City, Tokyo, 192-8577 Japan; 3 Database Center for Life Science, Research Organization of Information and Systems, 178-4-4 Wakashiba, Kashiwa, Chiba, 277-0871 Japan

**Keywords:** Databases, Glycomics

## Abstract

Glycans are known to play extremely important roles in infections by viruses and pathogens. In fact, the *SARS-CoV-2* virus has been shown to have evolved due to a single change in glycosylation. However, data resources on glycans, pathogens and diseases are not well organized. To accurately obtain such information from these various resources, we have constructed a foundation for discovering glycan and virus interaction data using Semantic Web technologies to be able to semantically integrate such heterogeneous data. Here, we created an ontology to encapsulate the semantics of virus-glycan interactions, and used Resource Description Framework (RDF) to represent the data we obtained from non-RDF related databases and data associated with literature. These databases include PubChem, SugarBind, and PSICQUIC, which made it possible to refer to other RDF resources such as UniProt and GlyTouCan. We made these data publicly available as open data and provided a service that allows anyone to freely perform searches using SPARQL. In addition, the RDF resources created in this study are available at the GlyCosmos Portal.

## Background & Summary

Various resources have created ad hoc databases since the *COVID-19*^1^ pandemic started, and research on its virus, *SARS-CoV-2*, is underway from various aspects. One of the findings so far is that many Spike proteins on the surface of the virus are covered with many glycans in order to cleverly avoid the immune system^2,3^. In fact, the *SARS-CoV-2* virus has been shown to have evolved due to a single change in glycosylation^4^. Furthermore, it has been found that glycans are deeply involved in binding to the human *ACE2* protein, which is a receptor found on the host^5^. Therefore, the information on glycans is closely related to the onset and symptoms of *COVID-19* and can be considered to be important in the development of vaccines, therapeutic agents, test kits, etc. in the future. However, at present, the research results of glycans related to viruses in general are not available in any format that can be read by machines and easily reused; many research results are found only as descriptions in the literature. Although databases containing those resources exist, proteins and pathways and symptoms related to *COVID-19* cannot be obtained efficiently because they have not been updated, are not comprehensive, or are provided only on web pages, and not stored in any database. Therefore, it is expected that more effective results can be obtained more quickly by developing a database that comprehensively and accurately provides relevant information to researchers. In particular, in order to accurately obtain the necessary information from diverse life science data, the Resource Description Framework (RDF) is attracting attention in this research field. By using RDF, data from heterogeneous resources can be integrated together with their semantics, thus enriching the data with meaning and allowing for inferences to be made upon the data. In this work, we used RDF to obtain integrated and comprehensive information related to pathogens and glycans in relation to human proteins, diseases, and pathways, together with their evidence.

In particular, we created an RDF database by obtaining data from three databases, PubChem^6^, SugarBind^7^, and PSICQUIC^8^. PubChem is a database focused on chemical compounds, but it has expanded its scope to also include genes, proteins, diseases and substances related to compounds. PubChem provides a part of their data in RDF format^9^ (PubChemRDF), but we added UniProt^10^ and gene information, which were only included in the Web pages but not in PubChemRDF, and connected them with disease resources and literature information. SugarBind is a database of pathogens that bind to glycans, and PSICQUIC is a service providing access to data from molecular interaction databases. Since both of these interaction-related databases were considered essential to link glycans and viruses, we recreated these datasets in RDF format. For SugarBind, information on diseases, glycans and ligand resources was RDFized with cross references to literature information. For PSICQUIC, interaction resources including the interactor and host organism were RDFized along with literature information. The PSICQUIC resources did not contain many interaction resources related to *SARS-CoV-2*. However, two interactions between P0DTC2, the UniProt ID for the *SARS-CoV-2 Spike protein*, and glycan structures were newly obtained.

**Fig. 1:**
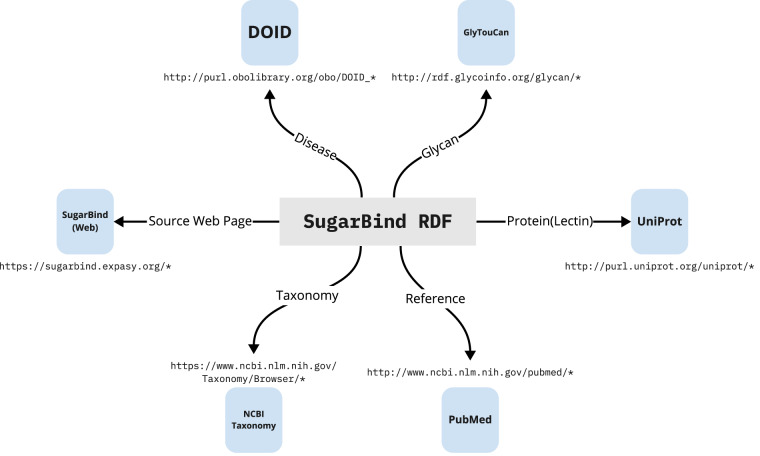
Links between SugarBind resources and other datasets. SugarBind RDF resources links to GlyTouCan ID, DOID, NCBI Taxonomy, PubMed, UniProt and SugarBind web interface. The SugarBind RDF resources are represented by gray squares, each arrow indicates what data each represents, and the referenced data resources are represented by a blue square.

In summary, we were able to establish a foundation for easy RDFization of glycans and their interactors based on the ontology we developed. Figure 1 illustrates how the RDF data based on SugarBind ontology enables the integration of data between diseases (DOID), glycans (GlyTouCan), proteins and lectins (UniProt), taxonomies and the literature. The interaction information derives from not only SugarBind, which focuses on glycans, but also PSICQUIC, which is a comprehensive resource of molecular interactions. Here, we show that this ontology is effective in representing the compound- and pathogen-interaction data in PubChem and SugarBind. In addition, by RDFizing PSICQUIC, we can now provide a unified means of access to major molecular interaction databases, and we expect that continued updates to these databases will increase the information on glycans and viruses. We made these data publicly available as open data for easy reuse and provided a service that allows anyone to freely perform searches using SPARQL (https://www.w3.org/TR/sparql11-query/) a standard query language for RDF data, at no cost. These resources are now available at the GlyCosmos Portal (https://glycosmos.org/) as well as its endpoint (https://ts.glycosmos.org/sparql).

## Methods

### RDF conversion of existing interaction data resources containing glycan, pathogen and diseases

#### SugarBind

**Fig. 2:**
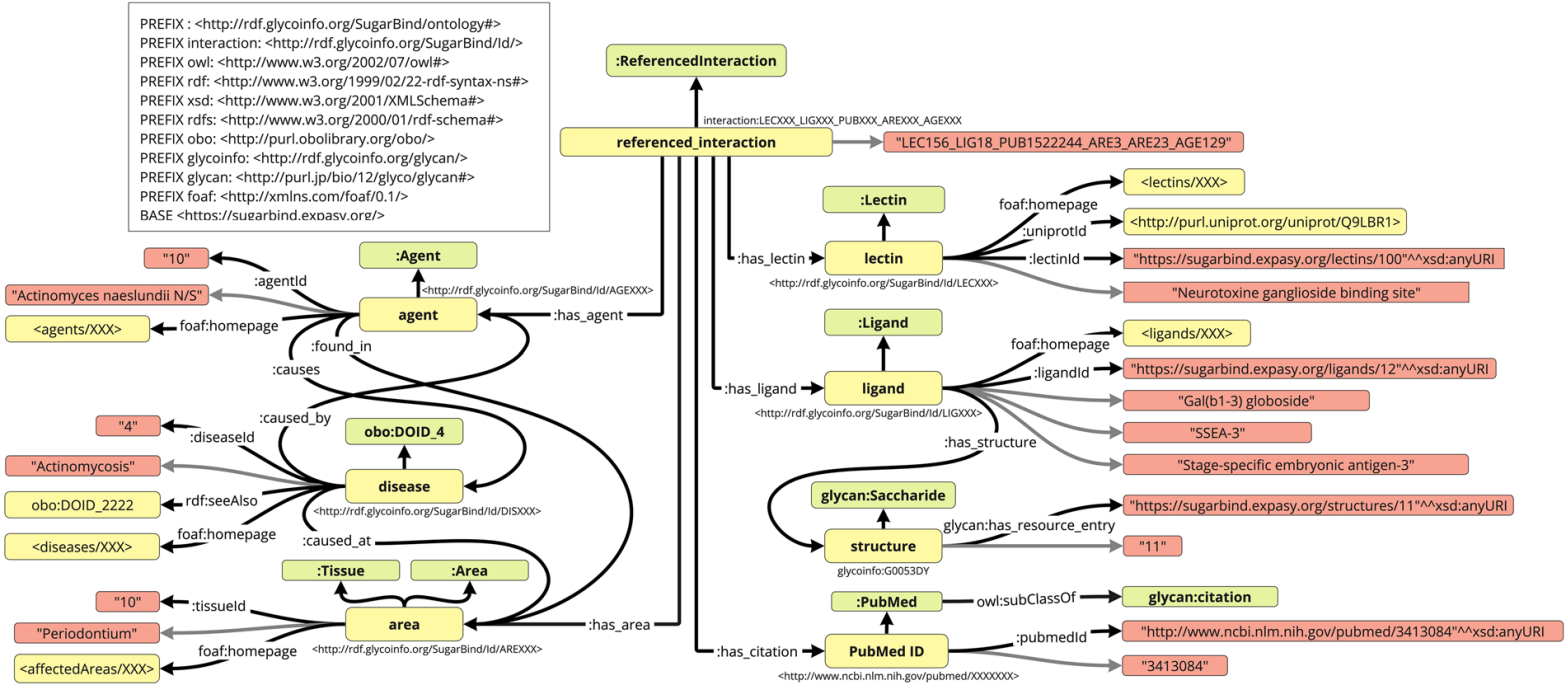
RDF schema for SugarBind resources. Glycan structure is defined by the GlyTouCan ID, the disease data are defined by the DOID, and the citation data are defined by PubMed. The classes (Green Square) and URIs (Yellow square) are displayed and they are connected by property represented by arrows. Black arrows without labels represent rdf:type, gray arrows represent rdfs:label.

First, we designed a new SugarBind ontology to represent the data in SugarBind. Previously, PAConto^11^ was developed as an ontology for the Pathogen Adherence to Carbohydrate Database (PACdb), which is a database of pathogens adhering to carbohydrates. Thus, we used it as a reference to construct the SugarBind ontology, which is easily connected to other related databases and has the same level of granularity as the data contained in the SugarBind database. Next, we obtained data on lectins, agents, diseases, ligands and structures from SugarBind to create the RDF data on glycan sequences that bind to pathogens. The script to retrieve SugarBind data was written in Python using BeautifulSoup (https://www.crummy.com/software/BeautifulSoup/bs4/doc/). The web URLs which were scraped are listed in Table 1. We generated csv files which contain the SugarBind data and designed an RDF schema (Fig. 2) to RDFize it. We then wrote Python scripts to convert the csv files into RDF. All programs used are available at https://github.com/glycoinfo/GlycanBind/tree/v1.0.2/SugarBind, and the SugarBind ontology has been registered into BioPortal at https://bioportal.bioontology.org/ontologies/SUGARBIND. All of the SugarBind resources were obtained on October 4, 2022.

**Table 1:** The URLs of the web pages used for scraped in the SugarBind resources are shown.

Resource	URL
Agent	https://sugarbind.expasy.org/agents?n=407
Ligand	https://sugarbind.expasy.org/ligands?n=204
Lectin	https://sugarbind.expasy.org/lectins?n=739
Area	https://sugarbind.expasy.org/affectedAreaTypes
Disease	https://sugarbind.expasy.org/diseases

#### PubChem

**Fig. 3:**
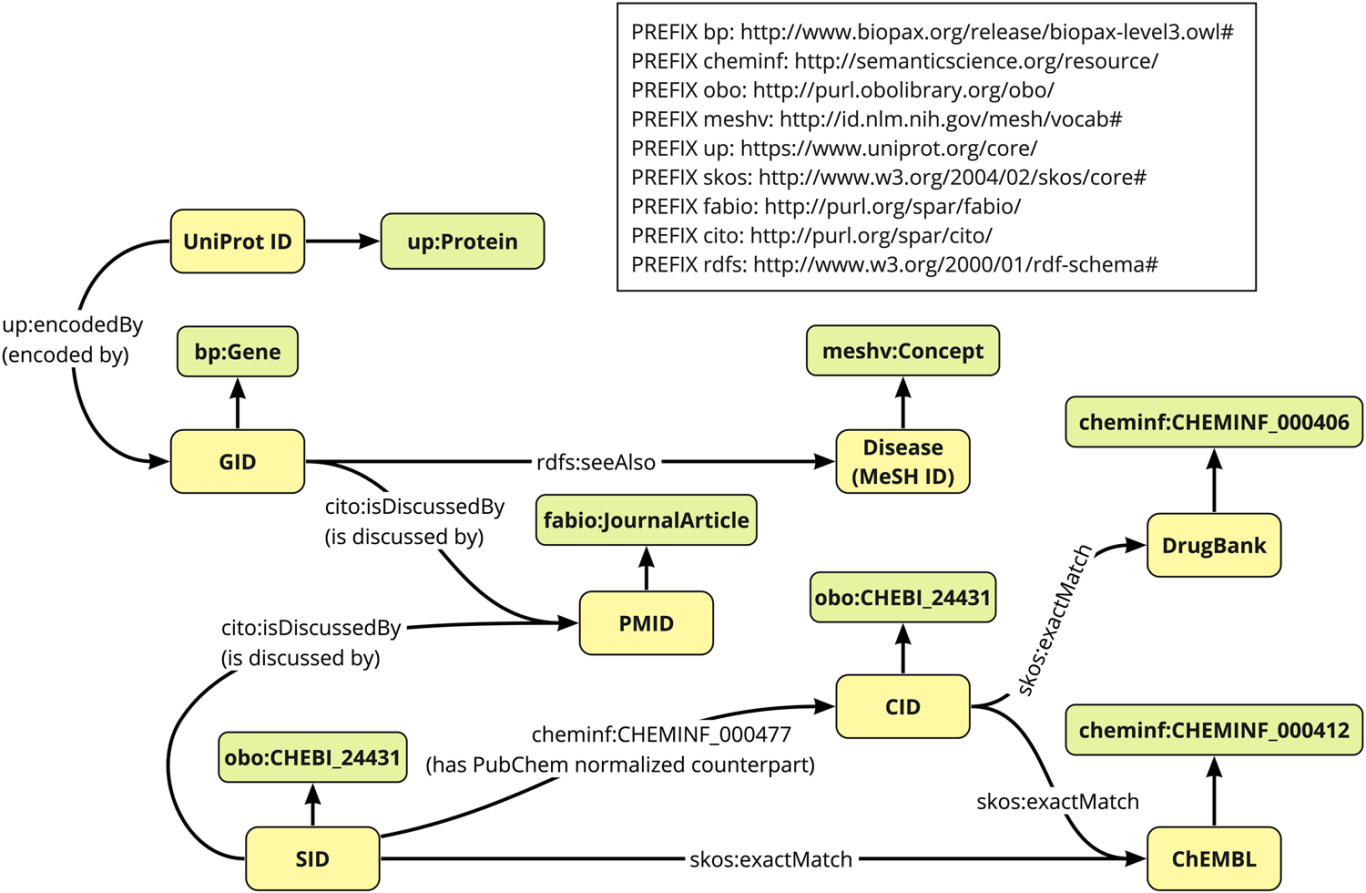
RDF schema for PubChem resources. The classes (Green Square) and URIs (Yellow square) are displayed and they are connected by property represented by arrows. Black arrows without labels represent rdf:type.

Some PubChem resources are already published in RDF format, called PubChemRDF. But all of the data that can be viewed through PubChem’s user interface is not included. Therefore, we worked on converting the *COVID-19*-related data on the PubChem Compounds pages, including the gene and UniProt data that are not included in PubChemRDF, to RDF. Since PubChem did not provide any application programming interfaces (APIs) to programmatically obtain these missing data resources, we used web scraping techniques to obtain them, resulting in 3 million pathway data sets related to genes and chemicals. The script to scrape PubChem pages was written in Python and uses the Selenium (https://www.selenium.dev/documentation/) library. Based on the obtained data, we created an RDF schema (Fig. 3) and converted the obtained data to RDF using RDFLib (https://rdflib.readthedocs.io/en/stable/) which is a Python package for working with RDF https://github.com/glycoinfo/GlycanBind/tree/v1.0.2/PubChem. All programs used to process the PubChem resources are available at. All PubChem resources were obtained on October 14, 2021.

#### PSICQUIC

**Fig. 4:**
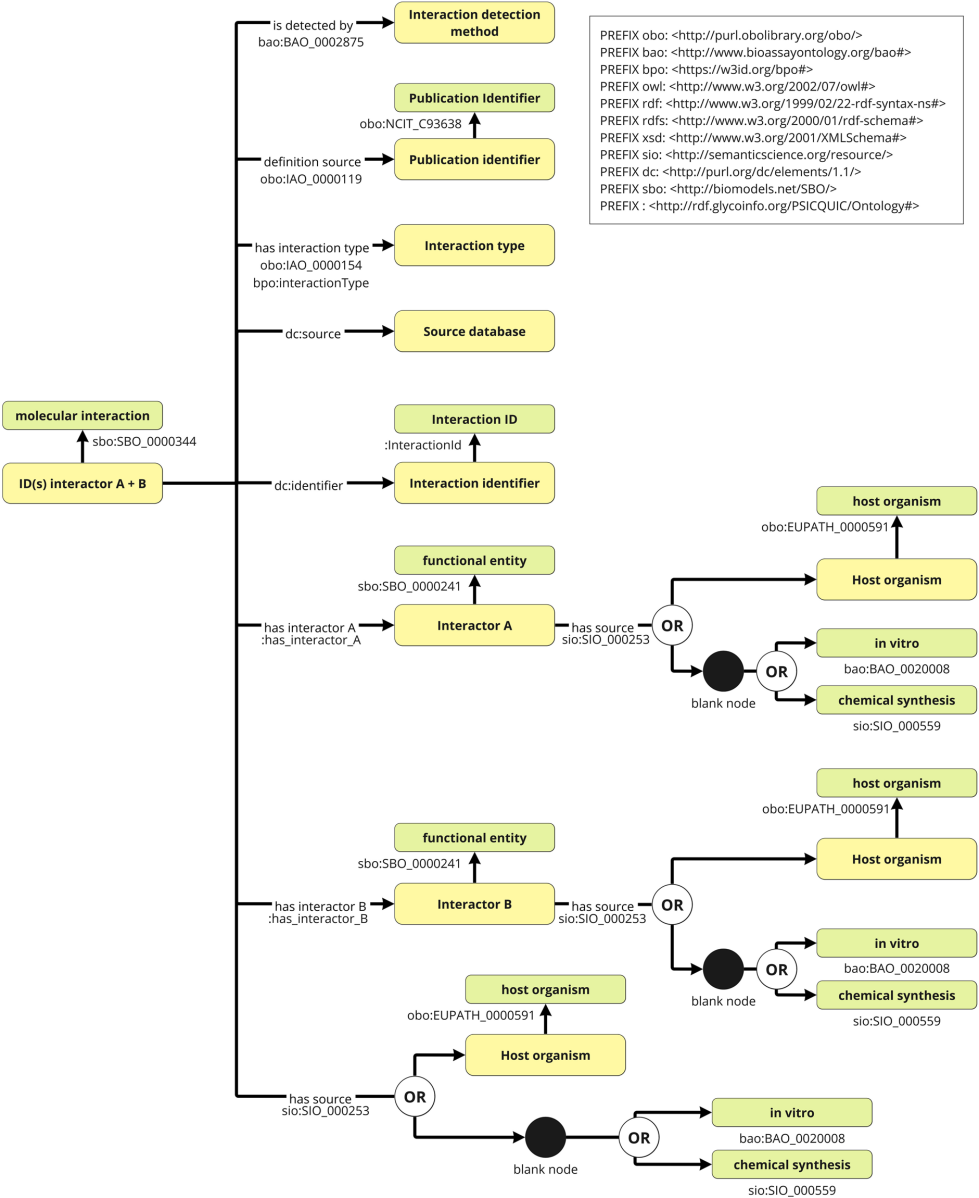
RDF schema for PSICQUIC resources. For PSICQUIC interaction data, MatrixDB.association from http://identifiers.org/ was, and linking it with the data for interactors, host organisms, and interaction types to be cross-referencing enabled. The classes (Green Square) and URIs (Yellow square) are displayed and they are connected by property represented by arrows. Black arrows without labels represent rdf:type, and ‘OR’ means that an object of a property having it on a branching arrow is either one of these pointed classes or instances.

We used the PSICQUIC REST API and retrieved data on human molecular interactions, which contains approximately 4.5 million data sets from 21 databases that were active in PSICQUIC as of January 29, 2022. Many of these interactions were related to protein-protein interactions, but also included chemical compound-protein interactions and compound-compound interactions. The chemical compounds could be mapped to GlyTouCan IDs if they are registered in ChEBI, so we attempted to retrieve as many human interaction data as possible. Based on this data, we created an RDF schema as shown in Fig. 4 and converted all data to RDF format. Interactor data in PSICQUIC are identified by various database IDs, which could be mapped into existing ontologies, but some could not be mapped. We thus tried to create URIs for some resources using identifiers.org. However, two scheme of http and https are used together depending on databases, and we consider it leads to discrepancies among URIs since we found several URI pairs that have only difference of http and https. So we used http://rdf.glycoinfo.org/dbid/ as the prefix URI to define URIs of interactor resources in a standardized format. The resulting list of prefixes, the data resources, and their counts are listed in Table 2. Sample python3 scripts to query the PSICQUIC REST API were provided by PSICQUIC at https://github.com/PSICQUIC/psicquic-solr-ws/blob/master/src/example/python/read-all-psicquic-python3.py. To filter the query to data related to glycans and involving *COVID-19*, “species:human OR species:9606 OR taxidA:human OR taxidA:9606 OR taxidB:human OR taxidA:9606” was set as the query option, and the data for the Interaction types were also set to psi-mi: “MI:0217” (phosphorylation reaction), psi-mi: “MI:0203” (dephosphorylation reaction) to exclude these interaction types to obtain glycan interaction data. Moreover, psi-mi: “MI: 1110” (predicted interaction) was also excluded to query only data obtained from experiments. The data obtained by this program were temporarily stored as tsv files. Next, we converted the tsv files to RDF according to our proposed schema using RDFLib. All source code used to query PSICQUIC using the REST API and to create RDF files are available at https://github.com/glycoinfo/GlycanBind/tree/v1.0.2/PSICQUIC.

**Table 2:** Prefix of all PSICQUIC interactor resources and the counts.

Prefix	Source Database	Count
http://rdf.glycoinfo.org/dbid/chembl	ChEMBL	101844
http://rdf.glycoinfo.org/dbid/CHEBI	ChEBI	88515
http://rdf.glycoinfo.org/dbid/uniprot	UniProt	50019
http://rdf.glycoinfo.org/dbid/ncbigene	NCBIGene	21601
http://rdf.glycoinfo.org/dbid/ensembl	Ensembl	5701
http://rdf.glycoinfo.org/dbid/refseq	RefSeq	5081
http://rdf.glycoinfo.org/dbid/intact	IntAct	3169
http://rdf.glycoinfo.org/dbid/rogid	Rogid	910
https://bioregistry.io/reference	Bioregistry	26
http://rdf.glycoinfo.org/dbid/insdc	INSDC	22
http://rdf.glycoinfo.org/dbid/reactome	Reactome	21
http://rdf.glycoinfo.org/dbid/pdb	PDB	11
http://rdf.glycoinfo.org/dbid/chembl.target	ChEMBL	7
http://rdf.glycoinfo.org/dbid/intenz	IntEnz	7
http://rdf.glycoinfo.org/dbid/rhea	Rhea	5
http://rdf.glycoinfo.org/dbid/complexportal	Complex Portal	1
http://rdf.glycoinfo.org/dbid/ensemblgenomes	Ensembl Genomes	1

### Loading of RDF data into virtuoso and integration into the GlyCosmos portal

All RDF data have been loaded into our Virtuoso RDF database, where users can make queries and obtain data through the GlyCosmos endpoint (https://ts.gslycosmos.org/sparql). The names of the graphs of all the resources is shown in Table 3. Moreover, we designed an API that can make queries using SPARQList (https://github.com/dbcls/sparqlist) which is middleware for providing a REST API that returns the results of SPARQL queries to clients. The GlyCosmos Web interface displays our RDFized data as searchable tables. The URLs of our resources are shown in Table 4.

**Table 3:** List of Graph name of PubChem, SugarBind and PSICQUIC.

Resource	URL
PubChem	http://rdf.glycosmos.org/glycovid_pubchem
SugarBind	http://rdf.glycosmos.org/sugarbind
PSICQUIC	http://rdf.glycosmos.org/psicquic

**Table 4:** List of GlyCosmos URLs of PubChem, SugarBind and PSICQUIC.

Resource	URL
PubChem	https://glycosmos.org/glycoproteins
SugarBind	https://glycosmos.org/sugarbinds
PSICQUIC	https://glycosmos.org/psicquics

## Data Records

The GlycanBind dataset, including original data, source code and generated RDF files, is available at Zenodo^12^ at 10.5281/zenodo.8072786. There are four folders contained in the dataset:

- SugarBind: This folder contains the scripts and data scraped from the SugarBind database as well as scripts to generate the RDF data. There are three subfolders:
  - img folder containing an image of the RDF schema.
  - ontology folder containing the SugarBind ontology.
  - shexer folder containing the program to use ShEx and validate the data and the results of evaluation.
- PubChem: This folder contains the many scripts and data scraped from the PubChem database as well as scripts to combine and generate the RDF data. There are three subfolders:
  - img folder containing an image of the RDF schema and of a sample SPARQL.
  - source folder containing the Python scripts developed for scraping the data.
  - shexer folder containing the program to use ShEx and validate the data and the results of evaluation.
- PSCIQUIC: This folder contains the scripts and molecular interaction data scraped from the PSICQUIC database. There are four subfolders:
  - img folder containing an image of the RDF schema.
  - mylib folder containing the Python scripts developed for scraping the data.
  - shexer folder containing the program to use ShEx and validate the data and the results of evaluation.
  - uri_list folder containing the program for analyzing the ShEx results and the resulting data files.
- scripts: This folder contains the SPARQL queries used to validate all of the data.

### RDF data converted from existing databases

#### SugarBind

**Fig. 5:**
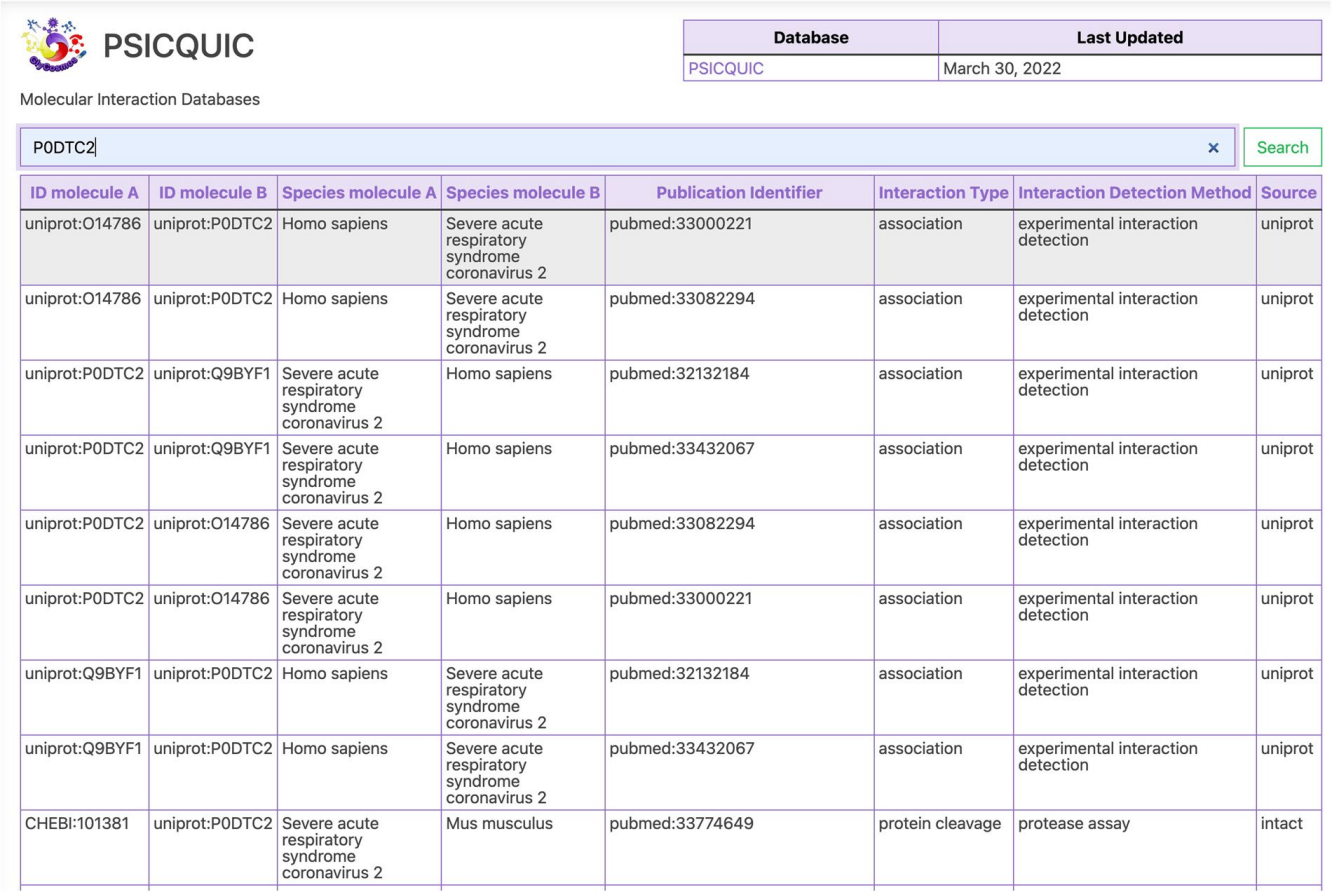
An example of how the GlyCosmos user interface can be used to search for COVID-19-related data. As a result of searching for the SARS-CoV-2 spike protein, 1,381,602 interactions were found as a result.

The current version of the SugarBind RDF resources we generated encompasses 16,675 triples, 739 interactions, and 35 properties consisting of 13 classes. All classes and the count of their objects are shown in Table 5. All the RDF data are in the Turtle format consisting of eight files. Almost all resources are defined using our SugarBind ontology, with several resources useing ontology provided by the Open Biological and Biomedical Ontologies (OBO) Foundry^13^, community development of interoperable ontologies for the biological sciences such as a National Cancer Institute Thesaurus (NCIt; https://ncit.nci.nih.gov/ncitbrowser/). The NCIT is a vocabulary for clinical care, translational and basic research, and public information and administrative activities; we used this to represent the SugarBind resource (Fig. 5). In addition, resources of glycan structure are defined using the Saccharide class in the GlycoRDF ontology^14^. Area, Organ, System, Tissue, and Cell classes are defined by the SugarBind ontology that describes affected area resources, which we created referencing SugarBind affected area type definitions. All SugarBind resources scraped from the SugarBind web pages have the predicate of foaf:homepage, and users can refer to SugarBind pages from their corresponding SugarBind resources. The SugarBind data created from SugarBind data are all linked to PubMed ID^15^ and DO IDs, so other resources linked to these IDs can be referenced from SugarBind resources. Moreover, glycan structure resources are all linked to GlyTouCan ID^16^, which makes it possible to reference the glycan resources in GlyTouCan and GlyCosmos, and other resources linking to the same GlyTouCan ID. Finally, SugarBind data were loaded into the GlyCosmos Virtuoso RDF database, so that it can be queried with SPARQL from the GlyCosmos endpoint (https://ts.glycosmos.org/sparql).

**Table 5:** Object count per its class in SugarBind RDF.

Class	Resource	Count
http://rdf.glycoinfo.org/SugarBind/ontology#ReferencedInteraction	Referenced interaction	739
http://rdf.glycoinfo.org/SugarBind/ontology#Lectin	Lectin	739
http://rdf.glycoinfo.org/SugarBind/ontology#Agent	Agent	407
http://rdf.glycoinfo.org/SugarBind/ontology#Ligand	Ligand	204
http://rdf.glycoinfo.org/SugarBind/ontology#PubMed	PubMed	179
http://rdf.glycoinfo.org/SugarBind/ontology#Area	Area	39
http://rdf.glycoinfo.org/SugarBind/ontology#Organ	Organ	16
http://rdf.glycoinfo.org/SugarBind/ontology#System	System	11
http://rdf.glycoinfo.org/SugarBind/ontology#Tissue	Tissue	9
http://rdf.glycoinfo.org/SugarBind/ontology#Cell	Cell	3
http://purl.obolibrary.org/obo/NCIT_C16785	Lectin	739
http://purl.obolibrary.org/obo/NCIT_C95009	Ligand	204
http://purl.obolibrary.org/obo/NCIT_C42881	PubMed	179
http://purl.obolibrary.org/obo/NCIT_C2991	Disease	46
http://purl.obolibrary.org/obo/DOID_4	Disease	46
http://purl.obolibrary.org/obo/NCIT_C12801	Affected area	39
http://purl.jp/bio/12/glyco/glycan#Saccharide	Glycan structure	173

#### PubChem

The current version of PubChem RDF resources encompasses 21,761,165 triples and 10 properties consisting of 7 classes. All classes and the count of the class objects are shown in Table 6. PubChem disease resources are defined using the URI http://id.nlm.nih.gov/mesh/vocab#Concept, which can then be referenced by other resources using MeSH IDs. In addition, disease resources using DO IDs can cross reference to the same disease resources in MeSH, so PubChem disease resources can also be linked. In order to test our PubChem RDFized data using the SugarBind ontology, we tried a SPARQL query to find commonalities between the PubChem and SugarBind resources based on Disease Ontology IDs in SugarBind and MeSH IDs (https://www.nlm.nih.gov/mesh/meshhome.html) in PubChem. As a result, 45 resources were linked and could be referenced; these were data related to *pneumonia*, *influenza*, *malaria*, and acquired *immunodeficiency syndrome*. This SPARQL query script has been published in https://github.com/glycoinfo/GlycanBind/blob/v1.0.2/scripts/sugarbind_doid_to_pubchem_meshid.rq. The created RDF data were loaded into GlyCosmos Virtuoso and can be queried with SPARQL from the GlyCosmos endpoint(https://ts.glycosmos.org/sparql).

**Table 6:** Object count per its class in PubChem.

Class	Count
http://biomodels.net/SBO/SBO_0000344	1826755
http://rdf.glycoinfo.org/PSICQUIC/Ontology#InteractionId	1374118
http://semanticscience.org/resource/SIO_000559	574302
http://biomodels.net/SBO/SBO_0000241	277275
http://purl.obolibrary.org/obo/NCIT_C93638	95731
http://www.bioassayontology.org/bao#BAO_0020008	7184
http://purl.obolibrary.org/obo/EUPATH_0000591	776

#### PSICQUIC

The PSICQUIC RDF resources we obtained encompasses 21,761,165 triples and 10 properties consisting of 7 classes. All classes and the count of the class objects are shown in Table 7. For PSICQUIC interaction data, MatrixDB.association from http://identifiers.org/ was used, and linking it with the data for interactors, host organisms, and interaction types could be cross-referenced. Using SPARQL queries with the created RDF data, we traced the ancestry of *SARS-CoV-2* (taxon:2697049) back to the genus level and searched for glycan data using the ChEBI IDs^17^ linked to the GlyTouCan IDs, which were registered as an interactors in PSICQUIC. However, no glycan structures were returned, most likely due to the fact that these databases have not annotated such data yet. This SPARQL query script is available at https://github.com/glycoinfo/GlycanBind/blob/v1.0.2/scripts/psicquic_sars_cov_2.rq. We also queried interactions between proteins and glycans. Glycan interactor resources were queried using the ChEBI ID linked to the GlyTouCan ID, and protein interactor resources were queried using the UniProt ID. As a result, 16 interactions were obtained, of which two were interactions between a glycan structure and P0DTC2, which is the UniProt ID for the *SARS-CoV-2 Spike protein*. This SPARQL query script is available at https://github.com/glycoinfo/GlycanBind/blob/v1.0.2/scripts/psicquic_glycan_interaction.rq. The created RDF data were loaded into GlyCosmos Virtuoso and can be queried using SPARQL from the GlyCosmos endpoint (https://ts.glycosmos.org/sparql).

**Table 7:** Object count per its class in PSICQUIC.

Class	Count
http://biomodels.net/sbo/sbo_0000344	1826755
http://rdf.glycoinfo.org/psicquic/ontology#interactionid	1374118
http://semanticscience.org/resource/sio_000559	574302
http://biomodels.net/sbo/sbo_0000241	277275
http://purl.obolibrary.org/obo/ncit_c93638	95731
http://www.bioassayontology.org/bao#bao_0020008	7184
http://purl.obolibrary.org/obo/eupath_0000591	776

### Data integration to GlyCosmos user interface

All the resources created in this project are available from the GlyCosmos user interface. GlyCosmos lists resources which were added in this study as a table, and the user can filter by keywords in the sidebar. As for SugarBind resources, GlyTouCan IDs are linked to GlyCosmos Glycan pages, so users can reference details of each glycan, which includes information such as the biosynthetic genes, core proteins, etc. In addition, the user can download these resources in csv, tsv, or json format. The GlyCosmos URL of the resources is listed in Table 4.

As an example of how the GlyCosmos user interface can be used to search for COVID-19-related data, Fig. 5 illustrates how the search for P0DTC2 lists 1,381,602 interactions pertaining to this protein, across not only human, but many other species, and with other proteins and chemicals in UniProt and ChEBI. Not only ACE2 (Protein ID Q9BYF1) but Neuropilin-1 (Protein ID O14786) show up as major interactors with the Spike protein. Moreover, chemical compounds such as Lipid A (CHEBI:134256) and aloxistatin (CHEBI:101381) have also been reported. All of the publications that have reported each of these interactions are also provided in the user interface.

## Technical Validation

We tried to validate the RDF data using Shape Expressions (ShEx) (https://www.w3.org/2001/sw/wiki/ShEx) generated by sheXer^18^. ShEx is a data modeling language that describes RDF nodes and graph structures. It can describe predicates and their associated cardinalities and datatypes, and it is effective for va lidating RDF data. In addition, ShEx can be automatically generated from a given RDF dataset by sheXer, which is a Python library, so we used it in our validation process. We generated ShEx on our RDF data, and the ShEx document generated by sheXer includes information on what properties and objects each class instance has, along with their statistics, as ShEx comments. These include the ratio of the number of instances having only one specific property to the total number of those having at least one of that property. Using this we, for example, can check whether or not there is an outlier having an extraordinarily large number of properties.

### SugarBind RDF resources

**Fig. 6:**
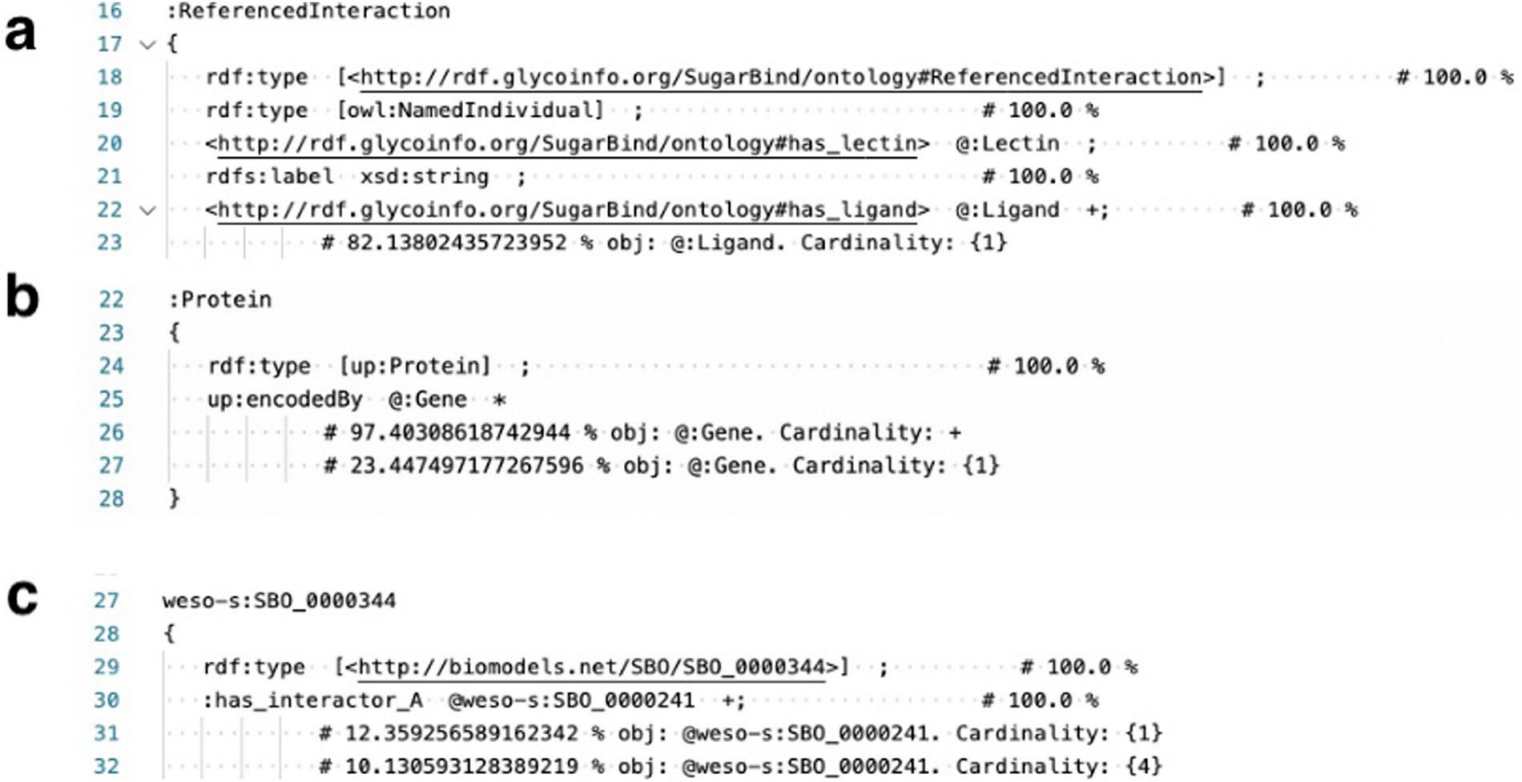
ShEx output by sheXer, indicating the integrity of each dataset. (**a**) ShEx data for ReferencedInteraction in SugarBind RDF. (**b**) ShEx data for Protein in PubChem. (**c**) ShEx data for molecular interaction in PSICQUIC.

A part of the resultant ShEx document on the SugarBind RDF data is shown in Fig. 6a. The data shown in the image are information on ReferencedInteraction, and the comment at the line 18 tells that 100% of the instances belong to the class of http://rdf.glycoinfo.org/SugarBind/ontology#ReferencedInteraction. As sheXer generates ShEx node constraints per class, this is obvious, and we checked the other property descriptions. We confirmed that on line 22, all (100%) of the ReferencedInteraction instances have one or more triples of http://rdf.glycoinfo.org/SugarBind/ontology#has_ligand as a property and instances of:Ligand as an object. Furthermore, on line 23, as a comment to ShEx, we could confirm that 82% have an only one instance of:Ligand. The validity of the data for other properties and class instances was similarly verified. All ShEx data for SugarBind RDF are available at https://github.com/glycoinfo/GlycanBind/blob/v1.0.2/SugarBind/shexer/shex_20221119164500.shex. The sheXer script to generate ShEx for SugarBind resources is available at https://github.com/glycoinfo/GlycanBind/blob/v1.0.2/SugarBind/shexer/main.py.

### PubChem RDF resources

Some of the results of the sheXer run on PubChem RDF are shown in Fig. 6b. The data shown in the image are information on Protein, that is, instances belonging to the class of up:Protein. We confirmed on lines 25 and 26 that 97.4% of all Protein instances have one or more triples of up:encodedBy as a property and instances of:Gene as a object. All ShEx data for PubChem are available at https://github.com/glycoinfo/GlycanBind/blob/v1.0.2/PubChem/shexer/shex_20221202112539.shex. The sheXer script to generate ShEx for PubChem resources is available at https://github.com/glycoinfo/GlycanBind/blob/v1.0.2/PubChem/shexer/main.py.

### PSICQUIC RDF resources

Some of the results of the sheXer run on PSICQUIC RDF are shown in Fig. 6c. The data shown in the image are information on Molecular interactions, that is, instances belonging to the class of http://biomodels.net/SBO/SBO_0000344. We confirmed that line 30 indicates that 100% of Molecular interaction instances have one or more triples of:has_interactor as a property and instances of http://biomodels.net/SBO/SBO_0000241 as an object. Furthermore, from the comments generated by ShEX on lines 31 and 32, we could confirm that 12% have only one instance of:Ligand and 10% have four instance of:Ligand. The validity of the data for other properties and class instances was similarly verified. All ShEx data for PSICQUIC are available at https://github.com/glycoinfo/GlyCovid/blob/main/PSICQUIC/shexer/shex_20221118100320.shex. The sheXer script to generate ShEx for PSICQUIC resources is available at https://github.com/glycoinfo/GlyCovid/blob/main/PSICQUIC/shexer/main.py.

## Data Availability

All code to generate the SugarBind RDF resources and the generated RDF data files are available from the GitHub repository as a v1.0.2 release at https://github.com/glycoinfo/GlycanBind/releases/tag/v1.0.2. The entire Github repository for the v1.0.2 release is archived on Zenodo^12^: https://zenodo.org/record/8072786.
